# Scalp Lesion as the First Manifestation of Pancreatic Adenocarcinoma, A Very Rare Case

**DOI:** 10.34172/aim.31235

**Published:** 2024-11-01

**Authors:** Luis Posado-Domínguez, Jonnathan Roldan-Ruiz, María Martin-Galache, Alejandra Ruiz-Villanueva, Maria L Perez-García

**Affiliations:** ^1^Department of Medical Oncology, University Hospital of Salamanca, Salamanca, Spain; ^2^Biomedical Research Institute of Salamanca, IBSAL, Salamanca, Spain; ^3^Department of Dermatology, University Hospital of Salamanca, Salamanca, Spain; ^4^Department of Internal Medicine, University Hospital of Salamanca, Salamanca, Spain

**Keywords:** Cutaneous metastases, Global assessment, Palliative care, Pancreas

## Abstract

Pancreatic adenocarcinoma is one of the most aggressive tumors. Its diagnosis is usually made in locally advanced or metastatic disease and survival is less than one year. The most frequent sites of metastatic involvement are the liver, peritoneum and lungs. Other organs such as the bones or the brain may be affected to a lesser extent. Cutaneous involvement of pancreatic adenocarcinoma is extremely rare with less than 150 cases reported in the literature since 1960. Most cases with cutaneous involvement involve the periumbilical region, in a lesion known as "Sister Mary Joseph’s Node". Scalp metastases are very rare and their diagnosis suggests advanced disease and the prognosis will be dismal. It is very important to perform a complete physical examination and a global anamnesis to guide the request for diagnostic tests. Once the diagnosis of pancreatic adenocarcinoma has been made, a global assessment will be necessary, involving different medical specialists, nurses, psychologists and social workers among others. In many cases, supportive care is the mainstay of treatment.

## Introduction

 Pancreatic adenocarcinoma is one of the most aggressive tumors. The diagnosis is usually made at a metastatic or locally advanced stage of the disease, making it impossible to use a surgical approach.^1^ First line treatment with the best chemotherapy schemes available, based on FOLFIRINOX or gemcitabine-Nab paclitaxel administration, provides an overall survival of approximately one year.^2^

 Regarding metastatic involvement, the most frequent sites of dissemination are the liver and peritoneum, followed by the lungs. This can lead to problems such as liver failure, intestinal obstruction due to carcinomatosis and respiratory failure due to carcinomatous lymphangitis. Cutaneous involvement of pancreatic adenocarcinoma is a very infrequent manifestation and a high clinical suspicion is necessary to focus the diagnostic study.^3,4^

 We analyzed the case of a 79-year-old woman with constitutional syndrome and skin lesions secondary to pancreatic adenocarcinoma.

## Case Report

 We report a 79-year-old woman with a history of hypertension, primary hypothyroidism, nonalcoholic liver disease, restless legs syndrome and type 2 diabetes mellitus.

 There was no family history of interest. The patient was independent for basic and instrumental activities, worked as a secretary, and was the main caregiver for her husband. She had no children, was an ex-smoker since 2004. She had IPA 10, and was a non-drinker.

 In November 2023, she consulted the Emergency Department for two months of persistent cough, associated in the last week with nausea, vomiting of food content, epigastric pain, and pain in the left shoulder. The patient reported a 5 kg weight loss in the last month, progressive asthenia until it became incapacitating, insomnia, and loss of appetite. The onset of diabetes in the last 6 months was also noted.

 Her vital signs were TAS 127/62 mm Hg, MAP 84 mm Hg, HR 105 bpm, 28 rpm, and SPO_2_ 96%.

**Figure 1:**
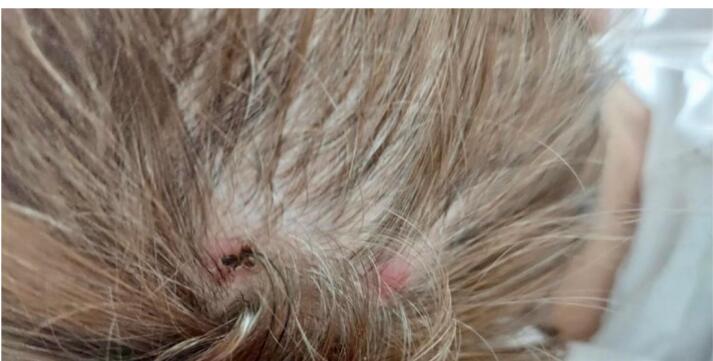
Photograph of the Patient’s Scalp. Papular, pinkish and irregular nodules, compatible with tumor lesion

**Figure 2:**
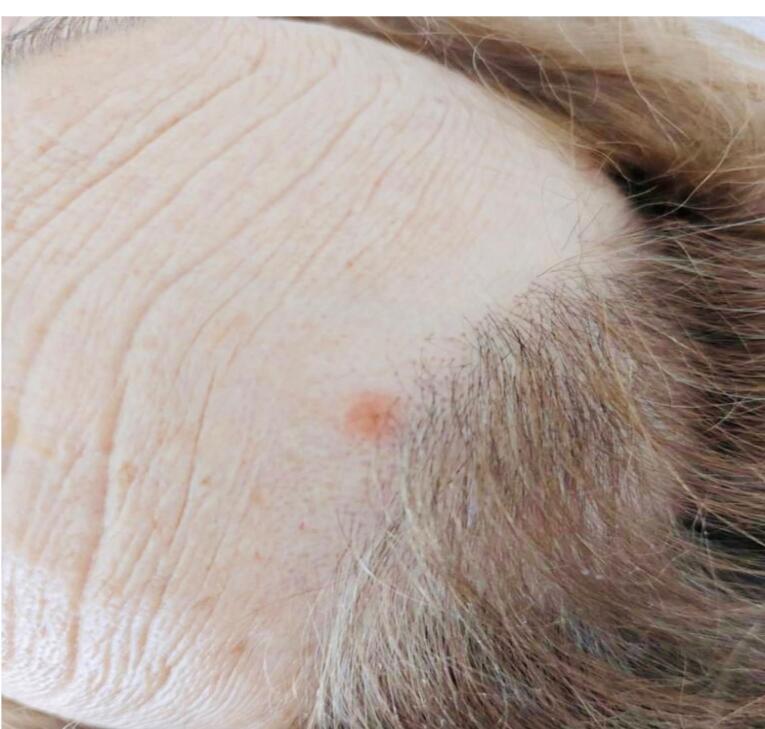
Pinkish Lesion on the Frontal Hairline of the Scalp, Compatible With Metastatic Involvement

 Examination revealed ECOG 3, Karnofsky 40 points, tachycardia, tachypnea, crepitation in the right lung base, moderate thinness (BMI 16.5), cutaneous-mucosal pallor and presence of four pinkish nodulopapular lesions on the scalp and frontal hairline with polymorphous vessel infiltrate and irregular lines, compatible with cutaneous metastases (Figures 1 and 2).

 Punch biopsy of the skin lesions and chest X-ray were performed with presence of interstitial infiltrate in the right pulmonary base.

**Figure 3:**
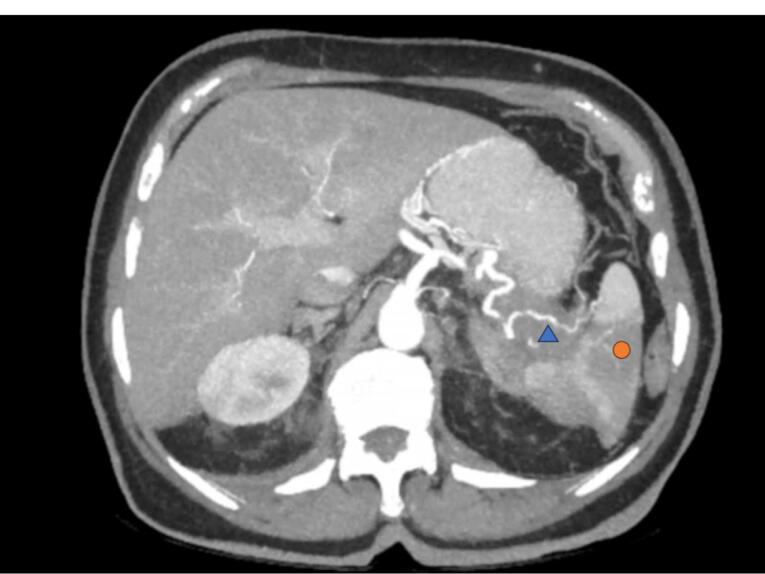
Hypodense and Poorly Defined Tumor in Pancreatic Tail of 5.8 x 4.7 x 2.7 cm (blue triangle). Hypodense area in the spleen suggestive of infarction (orange circle)

**Figure 4:**
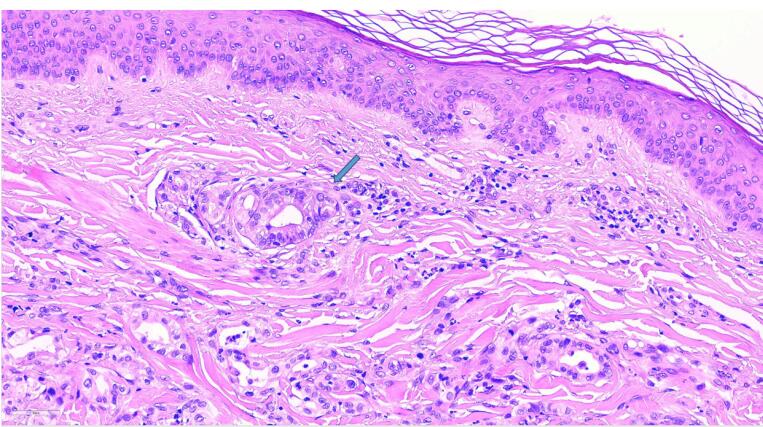
Hematoxylin-Eosin Staining of Scalp Biopsy. Atypical cells forming nested glandular structures are observed, compatible with adenocarcinoma (blue arrow)

 Investigations continued with a thoracic abdominopelvic CT scan with the finding of pancreatic tail neoplasia with associated splenic infarction, left adrenal and gastric infiltration together with associated carcinomatous lymphangitis (Figure 3). Laboratory analysis found a tumor marker CA 19.9 of 127324.4 KU/L. Skin biopsy showed metastasis of adenocarcinoma with phenotype CK7 + , CK20-, CDX2 + weakly focal compatible with pancreatic origin (Figure 4).

 She was evaluated in a multidisciplinary way by internal medicine, oncology, social work, psychiatry and palliative care unit (PCU) in order to optimize symptomatic and supportive treatment. After 17 days, she died in the PCU.

## Discussion

 In Spain, pancreatic cancer ranks 12th in incidence and 3rd in mortality. Among the factors that make it a poor prognosis neoplasm with a 5-year survival rate of less than 10% are late diagnosis due to vague and non-specific initial symptoms such as abdominal pain, asthenia or alterations in stool habits; its location, being surrounded by organs and blood vessels to which tumor cells can easily spread, and biological factors such as an unfavorable tumor microenvironment, in which stromal tissue abounds, preventing adequate oxygenation and protecting tumor cells from the arrival of cytotoxic agents.^1,3^

 Pancreatic cancer frequently metastasizes to the liver and peritoneum, followed by the lungs, bones and brain.^4,5^ Skin metastases are rare, accounting for less than 3% of the total.

 Most cutaneous metastases of pancreatic cancer described in the literature are periumbilical lesions, commonly referred to as “Sister Mary Joseph nodule”.^3-5^ Other skin areas to which it can metastasize are the neck, thorax, epigastric region and axillae. Several authors such as Cassalia et al, Zhou et al and Gómez-Díez et al highlight the rarity of pancreatic metastases in the scalp, with less than 140 cases documented in the literature.^3-5^ This type of lesions can simulate, in appearance, others of non-neoplastic etiology such as pyoderma gangrenosum, granulomas or keloids.^4^

 In the two main series of cutaneous metastases of pancreatic cancer, 44.2%‒76.2% of the tumors originated in the body and tail of the pancreas. The predominant subtype was adenocarcinoma (77%‒84.1%) and the male-to-female ratio was 1.6:1 and 1.3:1. It is uncommon for pancreatic metastases to be the only site of distant disease. In the study by Zhou et al, only 3 patients (7%) manifested with skin lesions without other involvement.

 The usual immunohistochemistry is positivity for CK7 and CK19. Negativity for CK20 is variable; however, it can be indicative of a specific diagnosis of pancreatic origin. The tumor marker CA 19-9 is elevated in more than 80% of cases.^3,4^

 CDX2 is weakly positive in approximately one third of pancreatic adenocarcinomas. In some tumors such as adenocarcinomas of gastric origin or ovarian carcinoma, the expression of this marker is associated with a better prognosis; however, some studies suggest that its positivity in tumors of pancreatic origin is associated with greater aggressiveness and mortality.^6^

 In the case we present, the positivity of CK7, negativity of CK20 and the marked elevation of CA 19-9 oriented the diagnosis towards a pancreatic origin, later confirmed by abdomino-pelvic thoracic CT.

## Conclusion

 Scalp metastases of pancreatic cancer are extremely rare and require a high clinical suspicion together with a thorough physical examination.

 Immunohistochemistry, CA 19.9 elevation and imaging tests allow the confirmation of the diagnosis avoiding more aggressive tests.

 The multidisciplinary approach is fundamental. In many cases, this finding implies an advanced diagnosis and the need to evaluate chemotherapy versus supportive care.
